# How are children with HIV faring in Nigeria?- a 7 year retrospective study of children enrolled in HIV care

**DOI:** 10.1186/s12887-015-0405-9

**Published:** 2015-07-22

**Authors:** Moyinoluwa A. Ojeniran, Abieyuwa Emokpae, Cecilia Mabogunje, Patricia Akintan, Moshe Hoshen, Ram Weiss

**Affiliations:** Lifescope Integrated Services, Lagos, Nigeria; School of Public Health, Hebrew University Hadassah, Jerusalem, Israel; Massey Street Children’s Hospital, Lagos, Nigeria; Clalit Research Institute, Jerusalem, Israel

## Abstract

**Background:**

To review the pediatric care and treatment program at Massey Street Children Hospital, in Lagos, Nigeria a retrospective analysis of medical records focusing on health services, survival and retention in care.

**Methods:**

The analysis covered a cohort of children initiated on antiretroviral therapy (ART) from 2005 to 2011. In this population, pediatric HIV care was defined as initiating ART between ages 0 and 14 years. Treatment initiation and follow-up were according to the Nigerian national guidelines for pediatric ART, which are based on World Health Organization guidelines adapted to our local context. The primary endpoint was mortality measured as cumulative survival. Other outcomes of interest included “loss to follow-up”, “transferred out”, and “stopped treatment”.

**Results:**

Mean (SD) age at ART initiation was 51 (39) months in female children and 52 (42) months in male children. After seven years of ART care, 64 % of the 660 study children were retained in care and on treatment, 16 % were lost to follow-up, 10 % were dead, and 9 % had discontinued HIV care at this facility for other reasons. World Health Organization disease stage, CD4 count, age, and year of ART initiation were highly predictive of mortality, while anemia at baseline was not statistically significantly associated.

**Conclusions:**

Overall study results suggest a viable pediatric HIV program exists at the study facility. Retention rates were lowest for the earliest cohort of infected children, which implies long-term challenges. Mother-to-child transmission programs need to be dynamic to stem the scourge of pediatric HIV in Nigeria.

## Background

Nigeria is the most populous country in Africa with an estimated population of 177,071,561 as of July 2013, including 3,229,757 persons living with Human Immunodeficiency Virus (HIV) [1]. This makes Nigeria the country with the second largest burden of HIV in the world. Of the 3.2 million children living with HIV globally, 91 % live in sub-Saharan Africa [2, 3], and 260,000 live in Nigeria [1]. Of the 860,000 deaths under age 5 in Nigeria in 2013, 70 % were due to HIV and other infectious diseases [1, 4]. Nigeria’s mother-to-child transmission rate is 27.3 % [1], and the country has the world’s highest burden of new HIV infections among children, which has declined only 19 % since 2009 [5]. Although prevention and elimination of pediatric HIV should remain high on the agenda, the antiretroviral needs of infected children should not be compromised [6]. Worldwide and in Nigeria, minimal emphasis has been drawn to children living with HIV. Of the 11.7 million people living with HIV in low- and middle-income countries who received antiretroviral therapy (ART) in 2013, only about 740,000 were children [7]. We need to draw attention to outcomes of children on pediatric ART and to ensure access to needed care for children with HIV globally.

Our study evaluated the follow-up of children with HIV in a dedicated clinic in Lagos, Nigeria. This clinic provides ART free of charge to all children seeking care and performs quarterly and semiannual follow-up to monitor treatment. We had three objectives: (1) to assess mortality in this vulnerable population of children with loss to follow-up and discontinuation of treatment as secondary outcomes; (2) to analyze the impact of baseline parameters at initiation of therapy (such as age, WHO stage, CD4, count and hemoglobin level) on mortality; and (3) to assess the ability of the clinic to retain treated children for continuous monitoring.

## Methods

### Study design

The study was an analysis of a cohort of children who began ART between 2005 and 2011. We used routine, paper medical records. Children were followed until they died (the primary endpoint) or the study ended.

### Setting

The study population was children receiving comprehensive HIV/AIDS care at Massey Street Children Hospital, an HIV care center for children that was one of the first such centers to offer free care in Lagos, Nigeria.

HIV care at the facility started in 2005 with support by Nigeria-approved President’s Emergency Plan For AIDS Relief (PEPFAR) funding from the United States Government. Treatment is free and based on national Nigerian guidelines [8, 9], which rely heavily on World Health Organization (WHO) guidelines. The guidelines specify the criteria for initiating and changing ART, the choice of ART, and routine monitoring.

According to the 2010 guidelines, infants aged 2–11 months and children aged 12–17 months required a positive DNA-PCR test before ART initiation unless a presumptive diagnosis of HIV was made. Children aged 18–24 months were required to undergo HIV rapid testing using the national testing algorithm. A child with confirmed HIV infection was started on ART if s/he:

- met criteria for WHO clinical stage 3 or 4 regardless of CD4 count or CD4 %;
- had CD4 count <750 cells/mm3 or <25 % in children aged 24–59 months with WHO clinical stage 1 or 2; or
- had CD4 count <350 cells/mm3 or <25 % in children >5 years with WHO clinical stage 1 or 2.

Before 2010 the recommended first-line ART regimen was a combination of stavudine, lamivudine, and nevirapine. Stavudine was replaced with zidovudine when the guidelines were revised in 2010 because of long-term toxicity in children. Children with severe anemia (Hb < 8 g/dl) before starting therapy were started on alternative ART regimens.

All recommended services were available in the hospital. Although a pediatric center, the hospital served all ages of HIV positive patients, with children being the point of entry for other family members. It also holds a unique mix of pediatric patients including both hospital-referred patients (via maternity and other wards) and self-referred families.

### Procedure

Data collection was carried out using a standardized data abstraction form administered at the HIV medical records section of the hospital. The practice allowed major indicators to be recorded in at least three different places, including the national ART register, child’s paper medical record, and the electronic platform, which was useful in checking consistency and validity of data. Because of space constraints and filing challenges, the most reliable source was the ART register, except for hemoglobin values. Where gaps existed, data were then checked with the paper or electronic record. A pediatric patient in the study was defined as a child 0–14 years old at the time of ART initiation.

The primary study outcome was mortality, and data on death were obtained when a child died in the hospital or when patients who missed visits were traced. The program traced patients by having a member of the people living with HIV support group contact their caregivers by phone or home visit. The primary source of data was the ART register.

To be included in the study, a child had to have confirmed or presumed HIV infection, be enrolled in the clinic, have started ART between 0 and 14 years of age, and have complete records. Diagnosis of HIV infection, which we obtained from the ART register, was based on Nigeria’s national guidelines. The WHO HIV stage at ART initiation and CD4 count, which was performed using a FACS Count Flow Cytometer, were also extracted from the ART register. Hemoglobin level at ART initiation, analyzed using an Immunotrol hematology analyzer, was derived from the patients’ medical records. Age was recoded into categories of 5 year bands. Within the 0–5 year old group, another variable was created which allowed separation of infants and children below 2 years from 2–5 year olds. This resulted in the following age bands: 0–2, 3–4, 5–9, and 10–14 years old [10]. To accommodate the gaps in age strata, a child above his age band by one month was included in the next age band.

The study received ethical approval from the Lagos State Health Service Commission. No informed consent was required from study participants.

### Data analysis

We conducted a univariate analysis using ANOVA with Bonferroni post hoc test to explore differences in variables across strata. A Kaplan-Meier univariate analysis which allows estimation of survival over time accounting for drop outs was used to compare mortality with study variables. Cox proportional hazard regression was used to model multivariable associations with survival, after testing the proportional hazard assumption by log(−log) plots. Analysis was performed using SPSS 20 for Windows (IBM Corp., Armonk, NY).

## Results

### Study population

During the study period 1434 (750 male and 684 female) children were confirmed as HIV infected and enrolled in HIV care. Of these children, 799 (55.7 %) started ART while aged less than 15 years, of which 660 (82.6 %) children (342 male and 328 female) were included in the study. Participants were followed for 29,624 person months. At the end of the study, 424 (64 %) children were still in care and treatment; 10.3 % started ART in 2005, 24.1 % in 2006, 14.4 % in 2007, 21.5 % in 2008, 12.7 % in 2009, 8.3 % in 2010, and 8.6 % in 2011. Recruitment dropped significantly after 2009 because of the establishment of additional centers and saturation at this center. At ART initiation, median values were: age 41 months, body weight 14 kg, CD4 count 552 cells/cubic millimeter, hemoglobin 10 g/dl, and WHO stage 3.

Baseline mean CD4 count at ART initiation varied by gender and age group (Table 1). Follow-up time was statistically significantly different between the 0–2 year old group, which had the shortest mean follow-up, and the other age groups. No statistically significant difference was observed for the 10–14-year age group compared with the 0–2; 3–4 and 5–9 year old groups. The year of ART initiation of the 0–2-year-old group varied significantly from that of the 3–4-year-old group, with more of the younger children initiating ART after WHO guidelines were recently revised. Mortality varied statistically significantly by age group, with poorer survival for the youngest children.

**Table 1:** Characteristics of study population by sex and age

*P* values for sex and age		Sex		Age			
		F	M	0 TO 2 years	3 TO 4 years	5 TO 9 years	10 TO 14 years
Age (months)	Mean (SD)	51 (39)	52 (42)	13 (6)	42 (11)	85 (17)	138 (14)
CD4 (cells/mm^3^) p*: 0.014; <0.0001	Mean (SD)	708 (623)	606(512)	801(607)	760 (541)	477(494)	268(385)
	Missing	17	12	16	4	7	2
Baseline who stage p: 0.944; 0.537	I: N(%)	30 (9.5)	37(10.9)	30 (12.9)	19(10.1)	16 (9.1)	2 (3.5)
	II : N(%)	84(26.7)	77(22.8)	54(23.3)	50(26.6)	40 (22.9)	17 (29.8)
	III: N(%)	191(61)	217(64.2)	142 (61.2)	115(61.2)	114 (65.1)	37 (64.9)
	IV : N(%)	9 (2.9)	7(2.1)	6(2.6)	4(2.1)	5 (2.9)	1(1.8)
	Missing	4	4	4	1	3	0
Outcome p^b^: 0.325; 0.003	^a^COT(N,%)	207 (65.2)	217 (63.5)	141(59.7)	130(68.8)	115(64.6)	38(66.7)
	D(N,%)	28 (8.8)	38(11.1)	37(15,7)	12(6.3)	11(6.2)	6(10.5)
	LTF(N,%)	53(16.6)	56(16.4)	43(18.2)	27(14.3)	32(18.0)	7(12.3)
	STOP(N,%)	2(0.6)	6(1.8)	4(1.7)	1(0.5)	3(1.7)	0(0.0)
	TO(N,%)	28(8.8)	25(7.3)	11(4.7)	19(10.1)	17(9.6)	6(10.5)
Follow up time (months) p: 0.484; 0.001	Mean(SD)	46(25)	44(25)	40(24)	49(24)	47(26)	45(25)
Baseline HB (g/dl) p: 0.463; 0.327	Mean(SD)	10.44(3.91)	10.19(3.86)	10.29(4.01)	10.75(4.88)	9.98(2.46)	9.90(2.89)
	Missing	71	83	65	34	43	12
Year of art initiation p: 0.848; 0.033	2005 (N,%)	32(10.1)	36(10.5)	17(25)	26(38.2)	22(32.4)	3(4.4)
	2006(N,%)	77(24.2)	82(24.0)	47(29.6)	52(32.7)	43(27)	17(10.7)
	2007(N,%)	50(15.7)	45(13.2)	26(27.4)	27(28.4)	32(33.7)	10(10.5)
	2008(N,%)	62(19.5)	80(23.4)	67(47.2)	33(23.2)	35(24.6)	7(4.9)
	2009 (N,%)	40(12.6)	44(12.9)	38(45.2)	26(31)	13(15.5)	7(8.3)
	2010(N,%)	28(9.1)	27(7.9)	24(43.6)	10(18.2)	14(25.5)	7(12.7)
	2011 (N,%)	29(9.1)	28(8.2)	17(29.8)	15(26.3)	19(33.3)	6(10.5)

*P values based on log-transformed CD4 values

^a^ *COT *Currently on treatment, *D* Death, *LTF* Lost to Follow up, *STOP* Stopped treatment electively, *TO* Transferred out to another facility

^b^Only Mortality was considered as outcome of interest in the ANOVA analysis

### Survival analysis

Cumulative survival was 81 % (SE 3.1 %) in children 0–2 years old at baseline, 92 % (SE 2.5 %) for ages 3–4, 92 % (SE 2.4 %) for ages 5–9, and 87.1 % (SE 5.0 %) for ages 10–14. Cumulative survival was 86.4 % (SE 7.4 %) for children with baseline WHO stage of 1, 93.1 % (SE 2.1 %) with stage 2, 86.3 % (SE 2.05) with stage 3, and 62.0 % (SE 14.0 %) with stage 4.

Overall mean survival time of the 660 study participants was 79.8, 95 % Confidence Interval (CI) 77.9 − 81.7 months. Mean survival time was 81.4 (CI 76.1 − 86.7) months for children with baseline WHO stage 1, 82.3 (CI 79.9 − 86.0) months for stage 2, 78.6 (CI 76.1 − 81.1) months for stage 3, and 59.5 (CI 41.4 − 77.6) months for stage 4, log rank p value = 0.003.

Mean survival times for children initiating ART was 74.9 (CI 71.0–78.8) months for 0–2 years, 83.0 (CI 80.2–85.7) months for 3–4 years, 82.9 (CI 80.0–85.8) months for 5–9 years, and 79.8 (CI 77.9 − 81.7) months for ages 10–14, log rank p value = 0.001.

Log rank P values for differences in mortality were 0.003 by year of ART initiation, 0.293 by anemia status at baseline, and 0.299 by gender. Overall, WHO stage and age had a statistically significant association with survival.

### Determinants of mortality

**Fig. 1:**
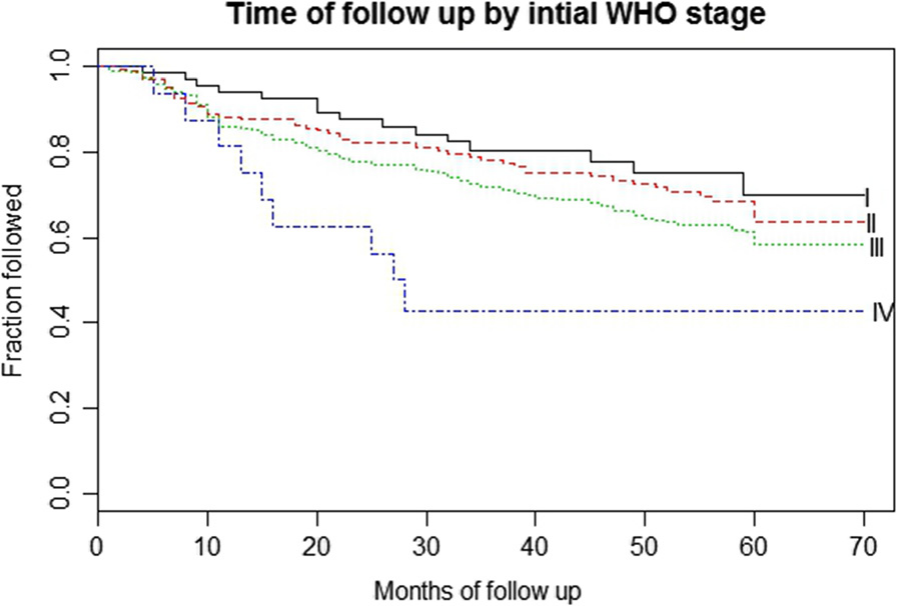
Kaplan-Meier survival by age at ART initiation

**Fig. 2:**
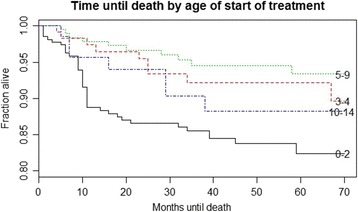
Kaplan Meier survival by baseline WHO stage

Children who initiated ART with more severe WHO disease categorization or younger age were less likely to survive (Figs. 1 and 2). In the same vein, the rate of death was less for children enrolled at younger ages and with higher CD4 counts (Table 2).

**Table 2:** Multivariable hazard ratio for death, among 0–14 aged HIV patients

	B	Sig.	HR (95 % CI)
Age	−0.019	0.002	0.981(0.969 0.993)
CD4	−1.174	0.000004	0.309 (0.188 0.509)
Gender	−0.227	0.252	0.797 (0.541 1.175)
Hemoglobin	−0.029	0.604	0.971 (0.871 1.084)

Appropriateness of model fitness was validated with a 2 Log likelihood test of model coefficients (overall *X* ^2^ = 28.360, *p* < 0.000011)

### Sensitivity analysis

Follow-up time for children censored because of loss to follow-up was evenly distributed across WHO stages at baseline (Kaplan-Meier *X*^2^ = 4.162, 0.245) and age (Kaplan-Meier *X*^2^ = 4.171, 0.244), which suggests the appropriateness of the survival analysis model. Also, missing values for baseline hemoglobin were not statistically differently distributed by age or sex (*X*^2^ = 5.588, 0.133; 0.347, 0.556), but in our study a child who died was less likely to have hemoglobin test results available (*X*^2^ = 43.907, <0.0001). CD4 counts were log transformed in the survival analysis to control for higher values in infants and young children.

## Discussion

This is a programmatic study of the impact of a government ART intervention program. In this setting, ART enrollment fluctuated over the years but consistently declined from 2009 to 2011. While Nigerian national treatment coverage for children has increased during 2005–2011 as the number of facilities providing HIV/AIDS care has also increased, our study demonstrates that individual facilities, especially those that have provided services for over 5 years, may be reaching a “saturation point” leading to declines in enrollment.

One of the findings in this study is the prediction of mortality by age and WHO stage in pediatric HIV patients, as we expected. Probability of survival was greatest for those commencing ART between age 2 and 9. Mortality was high in children under 2 years and children above 10 years and for children at WHO stages 3 and 4. As expected, lower hemoglobin values were associated with higher mortality although the association was not statistically significant, possibly because of missing values for this study variable. CD4 counts (log-transformed to allow sensitivity to extreme values for children under 5 years) and age (as continuous covariates) were significantly predictive of mortality using a multivariable Cox regression analysis model. Baseline hemoglobin values (due to missing values) and sex were not significantly predictive of mortality.

Exploring age and gender differences in baseline characteristics using ANOVA resulted in statistically significant differences by age group for CD4, mortality, and year of ART initiation. As we expected, CD4 values in the 0–2 and 3–4-year-olds were significantly different from the other groups, but not from each other because of the rapid physiologic changes in this age group. WHO stage was not significantly different by age as median WHO stage at ART initiation was 3 regardless of age. Children in this population were at an advanced stage of HIV before commencing ART. This might be because interim and national guidelines had recommended initiating ART for all patients aged 12 months with confirmed HIV infection regardless of WHO stage, CD4 count, or viral load since 2008. ART has shown tremendous improvements in short term outcomes in survival for children with HIV in Africa [11, 12]. Overall, ART led to significant reduction in mortality which can reach 50 % in children below 2 years without any intervention [13]. After ART initiation, mortality in our study was similar to other studies in both similar and diverse settings: 2.67 per 100 child years in our study vs. 2.31 per 100 child years in China [14] and 10 % in our study vs. 7.7 % in South Africa [11]. However, mortality rates should be interpreted with caution when loss to follow-up is excessive [15].

Massey Street Children Hospital offers the family-centered model of care in which an ‘index’ patient, in this case a child, is the entry point for other family members in need of care. Few studies have documented the impact of this model on child outcomes [16], probably because comprehensive HIV care is now widespread with index patients being adults, pregnant women, or children. However, barriers to care still hinder many people living with HIV and interfere with retention in care [17].

Median age at ART initiation in our study was 41 (inter-quartile range 18–77) months, which is similar to [18] or younger [16, 19, 20] than the median age found in other pediatric HIV studies in Africa and elsewhere [21]. The hospital is predominantly a children’s center and receives early referrals from other hospitals, which is most likely the cause of this age discrepancy. There is an opportunity to examine the impact of early intervention in this cohort of children because it is associated with better prognosis through immune and growth recovery. Strengthening the prevention and/or elimination of mother to child transmission program encourages early detection of infection and subsequent enrollment in HIV care [22]. Globally there are significant shortfalls and inequities in pediatric vs. adult HIV care leading to treatment gaps [1]. These disparities need to be addressed, and more sites should be opened or services should be effectively decentralized to other levels of care. However, decentralization of pediatric HIV care is fraught with concerns about quality of care, loss to follow-up, and drug resistance; studies reporting high success rates with decentralization [23, 24] have employed measures which are often not generalizable to other settings.

Overall participant retention rate in our study was 64 %, which is lower than but comparable to the 76.8 % rate (25) in a recent study conducted across purposively sampled sites with dedicated clinical staff for pediatric HIV. However, only about 50 % of the earliest cohort (2005) in our study were still on treatment at the end of 2011.

This analysis is one of the first in Nigeria to assess long-term outcomes of pediatric HIV care and uses routine program real-world data as opposed to trial or study data. The children in our study center include a rich mix of both hospital and self-referred patients which makes the interpretation of our study results appropriate for a wider local and international audience.

Limitations of our study include loss of patients to follow-up, which is a recognized potential confounder in chronic care. This factor was explored in the sensitivity analysis before any deductions were made. We have shown that this was not a major cause of bias. Selection bias for access to the ART program between clients delivering at health facilities and those delivering at home has been explored as an outcome of interest, i.e., association of age with survival. Missing data might also constitute a selection bias. Efforts were made to fill missing gaps with other sources of data. Because hemoglobin data were significantly missing, our analysis compared the characteristics of participants with missing data vs. other participants. We found that a child who died was less likely to have hemoglobin test results available. Thus, we propose that anemia might have been found to be a risk factor for mortality in this study population if the data had been available. Other biases such as subject error (e.g., recall of date of birth), instrument error (malfunction or calibration errors of measurement), or observer error (errors in recording or transferring data) are probably non-differential. Routine efforts in the HIV program minimize these errors through internal and external quality assurance, capacity building, and review meetings.

In Nigeria, the HIV epidemic is widespread and stable, and there are plans to scale up decentralization of services to primary care level to reduce inequalities and sustain access to care, especially in HIV infected children, who are especially vulnerable. An organized health care system was identified as one of the reasons for success in China’s pediatric HIV program, which has very low losses to follow-up [25]. High quality HIV care has also led to success of pediatric HIV programs in Nigeria [26]. However, the extent to which quality of HIV care in Nigeria is limited by political instability, poverty, and overstretched health workers should be explored before a reliable conclusion is made. It is essential that HIV programming in high prevalence settings continue to take up holistic strategies to create an organized system of care which ensures that children initiated in ART are retained. More importantly, elimination of pediatric HIV is imperative, as the burden of HIV on children, their families, the health system, and the nation as a whole is immense.

## Conclusions

Our study results show a high survival rate in an HIV program at Massey Street Children Hospital in a low/middle-income country, with a longer follow-up than is typically available in other studies. The main concern is to retain children in care after HIV diagnosis and, relevant to the study, after ART initiation.
